# Potential Diagnostic Hemorheological Indexes for Chronic Kidney Disease in Patients With Type 2 Diabetes

**DOI:** 10.3389/fphys.2019.01062

**Published:** 2019-08-20

**Authors:** Hoyoon Lee, Wonwhi Na, Sang Bae Lee, Chul Woo Ahn, Jun Sung Moon, Kyu Chang Won, Sehyun Shin

**Affiliations:** ^1^School of Mechanical Engineering, Korea University, Seoul, South Korea; ^2^Department of Internal Medicine, Yonsei University College of Medicine, Seoul, South Korea; ^3^Division of Endocrinology and Metabolism, Department of Internal Medicine, Yeungnam College of Medicine, Daegu, South Korea

**Keywords:** diabetic nephropathy, chronic kidney disease, deformability, critical shear stress, diagnosis

## Abstract

Many studies have demonstrated that an alteration in hemorheological properties is closely correlated with diabetic microcirculatory diseases. However, most of these studies have been limited to animal studies or used a small number of clinical samples, due to a lack of effective point-of-care (POC) devices to measure such properties within clinical environments. Owing to recent developments in microfluidic technology, several hemorheological POC devices have been designed that allow for the possibility of conducting extensive clinical studies using hemorheological measurements. Here, we reviewed recent clinical studies of diabetic kidney disease (DKD) associated with hemorheological parameters. We found that RBC deformability alone did not show a significant difference according to the degree of DKD, whereas critical shear stress (CSS) was found to be closely related to the ratio of albumin to creatinine and glomerular filtration rate. We also reviewed studies that alteration of hemorheological properties are associated with the development of DKD, which showed that CSS could be considered as a potential index to diagnose other diabetic complications as well as DKD.

## Introduction

The exponential increase in the incidence of diabetes is a major global healthcare issue (Bommer et al., 2017). In 2015, it is estimated that 8.8% (415 million) of the world’s population between the ages of 20 and 79 are suffering from diabetes, and the incidence of such diabetes is expected to increase by 1.5 times in 2040 (Baik, 2019). Diabetic vascular complications are a major cause of death in type 2 diabetic patients. Moreover, such diabetic complications lead to kidney failure, blindness, and non-traumatic limb amputation in adults (Afkarian et al., 2016). Diabetic complications in small blood vessels consist of diabetic eye disease known as DR, DKD, and DPN. These diabetic microangiopathies are closely correlated with macro-angiopathy such as coronary artery disease and stroke (Forbes and Fotheringham, 2017). Therefore, early detection of diabetic microangiopathy including DKD is crucial to prevent a progression to various complications and reduced quality of life of patient (Park, 2014).

Stages of kidney disease can be determined by the measured or estimated GFR. Stage 1 indicates normal value of GFR (>90 ml/min/1.73 m^2^) and stage 2 is only minimally reduced in GFR (60–89 ml/min/1.73 m^2^). The characteristics of stage 3 include the presence of microalbuminuria, a moderately reduced GFR range (30–59 ml/min/1.73 m^2^), and elevated blood pressure. Stage 4, known as clinical nephropathy, involves focal glomerular sclerosis and macroproteinuria, with a deterioration of the GFR (15–29 ml/min/1.73 m^2^). At stage 5, the GFR continues to decline (<15 ml/min/1.73 m^2^) and end-stage renal disease may develop (Levey et al., 2002).

Without treatment, DN has been reported to develop undetected, until noticeable symptoms occur. If DN is diagnosed early and managed appropriately, the progression of DN can be prevented or delayed (Shlipak, 2010). Several methods for screening DN have been introduced, with the urinary albumin-to-creatinine ratio (uACR) being the most commonly used clinical method to detect early DN. A uACR range between 30 mg/g and 300 mg/g of creatinine confirms a diagnosis of microalbuminuria, and has been reported to be excellent predictor for developing DN (Mogensen, 1984). Even though the uACR is relatively simple to use, inexpensive, and convenient for making determinations, it has several limitations as a screening tool for DN, including daily fluctuations in creatinine levels and being strongly influenced by other comorbidities such as infection, fever, menstruation, and exercise within 24 h, independent of kidney damage. Therefore, efforts continue to determine a more stable, complementary screening tool for early DN detection.

There have been numerous studies on alteration of hemorheological properties associated with DM since diabetic vascular diseases have shown close correlations with hemorheological indexes (Cho et al., 2008; Lee et al., 2018). Typical hemorheological indexes include deformability and aggregation of RBCs, ESR, CSS, yield stress and whole blood viscosity, These indexes had been only measurable in the laboratory for a long time. However, due to limitations in point-of-care technology (POCT) and the relevant instruments, the number of clinical patients involved in previous studies has not been large, and differing results have been reported. Owing to recent developments in POCT concerning hemorheological measurements, several more targeted clinical studies have been conducted within the last 5 years.

However, recent advances in leading-edge technologies including microfluidics have made the measurement of these hemorheological indexes possible in clinical settings. Thus, clinical studies have been undertaken to confirm an association between hemorheological alteration and diabetes-related kidney diseases (Lee et al., 2015, 2018; Chung et al., 2018). These clinical study focused on RBC deformability and CSS among hemorheological properties, since these two indexes had been the most potential diagnostic indexes of DM associated microangiopathy (Razavian et al., 1992; Brown et al., 2005). Therefore, this review aimed to assess the recent clinical studies concerning DN that had used hemorheological measurements. This is the first review that evaluates the association of CSS and DKD.

## Impact of Diabetic Mellitus on Hemorheology

Diabetes-related micro-vascular diseases have been shown to be closely associated with alteration of hemorheological properties such as increased blood viscosity (Monica et al., 2019), reduced cellular deformability (Brown et al., 2005), and increased cellular aggregation (Foresto et al., 2000). In addition, hemorheological alterations are frequently observed in the early onset of diabetes (MacRury and Lowe, 1990), and it has been suggested that these altered hemorheological properties may precede the growth of diabetic vascular complications (Martinez et al., 1998). Prior to focusing on a specific diabetic disease, it would be better to review the impact of DM on RBC rheology.

### Impaired RBC Deformability in Diabetic Mellitus

**FIGURE 1:**
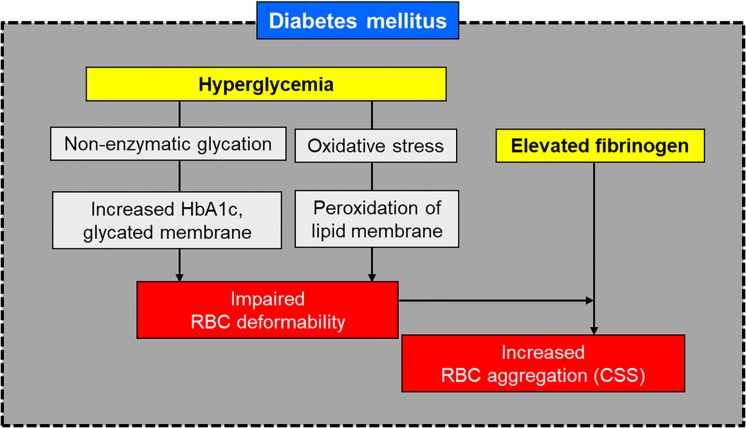
Schematic of hemorheological alteration in diabetes mellitus.

When insulin control fails, there is a consequent increase of glucose level in the blood plasma, which leads to hyperglycemia. Hyperglycemia is a major metabolic perturbation that results in microvascular damage in patients with diabetes (Friedman, 1981; Porte and Schwartz, 1996). However, the mechanism through which hyperglycemia leads to DN is not well understood. It has been reported that non-enzymatic glycation and oxidation (glycoxidation) forms AGEs, which is closely related to the pathogenesis of DKD (Makita et al., 1991; Fukami et al., 2008; Singh et al., 2014). Also, glycated hemoglobin (HbA1c), which represents glycation of RBCs, showed inverse correlation with RBC deformability in DM (Keymel et al., 2011) and DN (Shin et al., 2007b). Figure 1 depicts schematically the influence of diabetes-related factors on RBC deformability and aggregation.

Reduced RBC deformability is frequently observed in T2DM. The reduction of RBC deformability is associated with the denaturation of the cell membrane through the effects of glycation and oxidative stress. An abnormal blood glucose level results in biochemical changes within RBC membrane proteins and membrane lipids. Oxidative stress, associated with high-level glucose concentrations, results in denaturation of the RBC membrane lipids and proteins (Resmi et al., 2005). Glycosylation of membrane proteins, which is closely related to reduced membrane fluidity (Watala et al., 1985), is the main cause of the reduction of RBC deformability (Schwartz et al., 1991; Symeonidis et al., 2001).

Also, the reduction of RBC deformability is associated with increased viscosity of glycosylated internal fluid. Many studies have concluded that denaturation of the RBC membrane and increased internal viscosity of erythrocyte induce abnormal viscoelastic properties of erythrocyte membranes and may contribute to the onset of diabetic vascular diseases (Watala et al., 1992; Linderkamp et al., 1999). Consequently, the adverse alteration of RBC deformability can degrade the primary functions of RBCs, including the transportation of metabolites through the blood vessels (Caimi et al., 1993; Gyawali et al., 2016).

### Increased RBC Aggregation in Diabetes Mellitus

Increased RBC aggregation is commonly encountered in patients with T2DM, which accompanies the change of composition in the erythrocyte membrane. RBC membrane is slightly negative charged due to the sialic acid of glycoproteins on RBC membrane. The negative-charge on the RBC membrane result in electrostatic repulsion between RBCs and consequently reduces erythrocyte aggregation (Rogers et al., 1992). However, many studies reported that there was a significant reduction in the negative charge on the membrane surface for patients with T2DM (Gambaro et al., 1988; Budak et al., 2004), leading to an increased RBC aggregation (Rogers et al., 1992).

Higher plasma fibrinogen levels have been found in patients with T2DM than in healthy controls (Mahendra et al., 2015). Fibrinogen production and its concentration in plasma increase in patients with insulin-resistant T2DM. Increased fibrinogen level in plasma is an important risk factor of cardiovascular disease in patients with T2DM. When hyperfibrinogenemia occurs alongside decreased albumin levels, RBC aggregation is further increased (Angelkort, 1999). Moreover, if high levels of fibrinogen correspond with impaired RBC deformability in patients with T2DM, there is likely to be a synergistic effect on the tendency of RBCs to aggregate (Xue et al., 2013).

Increased RBC aggregation is considered to be the main cause of diabetic vascular complications since strongly aggregated RBCs cannot flow through capillaries (Grigoleit et al., 1973). More studies have provided supporting data on the relationship between hyperaggregation of RBC and hemodynamic resistance *in vivo* (Yalcin et al., 2004). Furthermore, patients with T2DM who had increased RBC aggregation showed a greater prevalence of peripheral vascular diseases than those without diabetes. Thus, it has been proposed that increased RBC aggregation is directly associated with this pathological results (Osmundson et al., 1990; Maser et al., 1991).

## Hemorheology

Hemorheology is the study of deformation and flow of blood and its cellular components. Since erythrocytes comprise the major components in blood, many hemorheological studies have focused on RBC rheology, such as deformability and aggregation of RBCs. Owing to the deformation and aggregation of RBCs, whole blood exhibits shear-thinning non-Newtonian viscosity. Viscosity of whole blood decreases with shear rate. This shear-thinning characteristic is mainly due to RBC aggregation as well as RBC deformability. RBC aggregation involves the auto-assembly of RBCs at low shear rates, whereas RBC deformability relates to shape changes under shear stress in liquid flow. When whole blood is under a high shear stress field, RBCs tend to disperse and deform. When shear stress gradually decreases, elongated RBCs return to their original shape and dispersed RBCs tend to aggregate at a certain shear stress, with the minimum stress required to prevent RBC aggregation known as the CSS of whole blood.

### RBC Deformability

#### Determinants of RBC Deformability

Deformability is a key feature of RBC that allows RBCs to pass through smaller vessels than themselves. At the cellular level, the major determinants of RBC deformability are (i) protein phosphorylation in membrane and cytoskeletal organization, (ii) control of ion and water of intracellular fluid (iii) surface area-to-volume ratio and (iv) the metabolism and integrity of hemoglobin (Kim et al., 2015; Huisjes et al., 2018). These factors are significantly altered in sickle cell disease and sepsis (Baskurt et al., 1998; Alapan et al., 2016). In addition, these factors are also closely related to adenosine triphosphate levels and the redox state (Karger et al., 2012; Kuhn et al., 2017). Combinations of these factors within hereditary and environmental conditions determine RBC deformability. This deformability regulates the blood’s life span as well as the efficiency of oxygen transport. A slight reduction in RBC deformability may result in decrease of blood flow into capillaries and subsequently result in various microvascular diseases (Shin et al., 2007b).

#### Measurement Techniques

A filtration method has been in use since the early days of measuring RBC deformability. This method involves assessing the capability of RBCs to pass through either small pores or microfluidic channels with applying positive or negative pressure (Reid et al., 1976; Bransky et al., 2007). RBC deformability (or rigidity) can be evaluated by measuring the passing time of a certain volume of RBCs through a filter. Even though the filtration method is simple, it has various problems including the blockage of filters by rigid leukocytes and the aggregates of activated platelets. Because of these limitations, filtration methods have produced variations in experimental results. There has been an attempt to mitigate these limitations using a mesh processed with micro-fabrication - (Odashiro et al., 2015).

**FIGURE 2:**
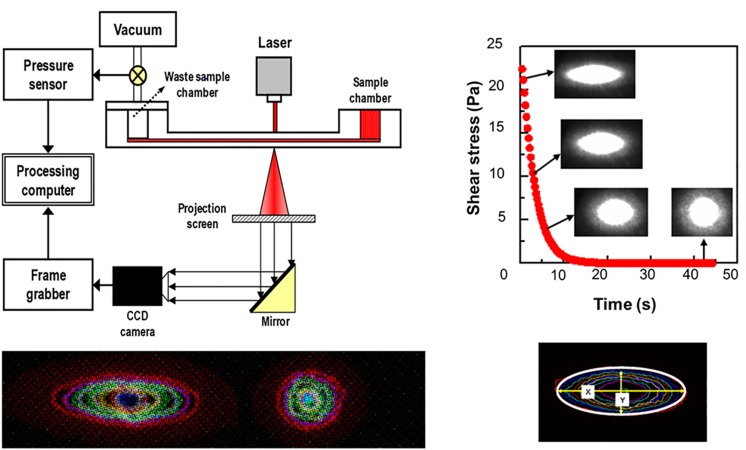
Schematic of microfluidic ektacytometry to measure red blood cell deformability (reproduced with permission from Shin et al., 2007a).

Laser diffractometry is another key method for measurement of cell deformability, because of its precision and reproducibility. There exist three commercially available laser diffractometers, adopting different shearing flow geometries. Their geometries are cop-and-bob, plate–plate and Poiseullie flow channel, respectively (Tomaiuolo, 2014; Kim et al., 2015). Among them, a microfluidic ektacytometry, which is designed for POC test, has been applied to measure RBC deformability for the diagnosis of patients with potential microcirculatory diseases (Shin et al., 2007b). The technique is performed on a shear stress-scanning laser-diffractometer (RheoScan), which measures an EI over an appropriate range of shear stresses [0.1–20 pascal (Pa)], as shown in Figure 2 (Shin et al., 2007a; Baskurt et al., 2009a). Microfluidic ektacytometry provides a maximum elongation index (*EI*_max_), an elongation index at 3 Pa (*EI*_3__Pa_), and half shear stress (*τ*_1__/__2_). The half shear stress is the value corresponding to shear stress where the *EI* is 50% of *EI*_max_ (Baskurt et al., 2009b). These parameters (*EI*_3__Pa_, *EI*_max_, *τ*_1__/__2_) are still being tested for their usefulness in diagnosis of various deformability-associated disorders. Table 1 summarized advantages and drawbacks of each measurement technique with operating principles.

**TABLE 1:** Determinants of RBC Hemorheology and measurement techniques.

	**Determinants**	**Measurement techniques**
Deformability	***Intrinsic factors*** - ratio of surface area to volume) - rheological properties of hemoglobin - membrane protein phosphorylation - cytoskeletal integrity. metabolism (Kim et al., 2015; Huisjes et al., 2018) ***Experimental alteration*** - variations in osmotic pressure, calcium, NO and temperature - membrane protein/lipid modification - Hb-Membrane interaction, - ATP-depletion (Singh and Shin, 2009)	***Filtration*** - membrane (Reid et al., 1976): - microfluidic filter (Bransky et al., 2007) - micromesh (Odashiro et al., 2015) - clogging, *laboratory, low reproducibility* ***Laser diffraction*** - cup-and bob geometry (LORCA, Hardeman et al., 1994): widely used - parallel disks (Rheodyn SSD, Schmid-Schönbein et al., 1996) - microfluidics (RheoScan-D^∗^, Shin et al., 2007a) - *not single cell analysis, widely used* ^∗^ disposable chip, convenient for POC test
Aggregation	***Cellular factors*** - Surface charge density (Nash et al., 1987) - membrane strain (Meiselman, 1993) - cell age (Rampling et al., 2004) ***Plasmatic factors*** - temperature, pH, osmotic pressure - protein constituents/concentrations - hematocrits (Ben-Ami et al., 2003; Lee et al., 2016b)	***Optics*** - cup-and bob, backscattering (LORCA, Hardeman et al., 2001) - Cone-and-plate, light transmitting (Myrenne Aggregometer, Kiesewetter et al., 1982) - Stirrer in a disk, light transmitting (RheoScan-A, Shin et al., 2009a) - *widely used*, arbitrary units of indexes
Critical shear stress, CSS (or critical shear rate, CSR)	***Critical shear rate*** (*s*^–1^) - Hct-independent (Hardeman et al., 2001) ***Critical shear stress*** (*mPa*) - Hct-independent (Razavian et al., 1992; Hou and Shin, 2008) ***Affecting factors*** - Similar to conventional aggregation indexes (Xue et al., 2013)	***CSR*** - cup-and bob, backscattering (LORCA) - dimensional index, hct-dependent, limited use in laboratory ***CSS*** - microfluidic, backscattering (RheoScan-AnD^∗^, (Shin et al., 2009b) - dimensional index, hct-dependent, adopting disposable chip, convenient for POC test

### RBC Aggregation

Similar to RBCs from most mammalian animals, human RBCs tend to aggregate forming of linear stacking shapes, which is often called rouleaux (Chien and Jan, 1973; Baskurt et al., 2012). One-dimensional forms of rouleaux can grow and form three-dimensional (3-D) networks. While these 3-D network aggregates grow exponentially from flow to stasis, a shear flow or mechanical shear can break aggregates into smaller aggregates and disperse them into individual cells (Shin et al., 2004). RBC aggregation and disaggregation are reversible processes that can occur in response to ambient shear forces, and aggregates will re-occur when external shear force is decreased (Shin et al., 2006). Thus, the degree of aggregation is determined by the balance of forces between the promoting aggregation and the opposing aggregation. Whereas the forces promoting aggregation are only partially understood through two conflicting theories, namely, polymer bridging and depletion layer theory (Neu and Meiselman, 2002). The factors affecting RBC aggregation can be divided into cellular and plasmatic factors (Rampling et al., 2004).

#### Cellular Factors

Cellular factors involve the intrinsic tendencies of RBCs to form aggregates, whereas plasmatic factors concern the nature of a suspending medium. RBC aggregation substantially differs from RBC aggregability, even though both terms are frequently conflated. RBC aggregability reflects the intrinsic tendency of RBCs at a cellular level to form aggregates, whereas RBC aggregation refers to the overall tendency of RBCs to aggregate. Since RBC aggregability is a cellular property promoting RBC aggregation, plasmatic factors such as the concentration of fibrinogen should be excluded in accessing RBC aggregability.

An assessment of RBC aggregability in different samples can be undertaken replacing autologous plasma with a standard medium (Meiselman, 2009). Water-soluble high molecular weight polymers, such as polyethylene glycol, polyvinylpyrrolidone, or dextran, are recommended as standard suspending media. Seventy kilodalton (kDa) dextran is frequently used for RBC aggregability comparison testing (Meiselman et al., 2007). Such measurements exclude the effects of donor-dependent plasma protein on RBC aggregation and thus distinguish cellular factors affecting RBC aggregation. Typical cellular factors are surface charge density (Nash et al., 1987) and membrane strain (Meiselman, 1993).

#### Plasmatic Factors

Concerning the plasmatic factors, there are various relevant plasma proteins and osmolality-affecting components. Plasma proteins affecting RBC aggregation have been found to be fibrinogen, albumin, and globulin (Yamamoto, 1986; Armstrong et al., 2004). Among them, fibrinogen has been shown to be the most important factor of RBC aggregation (Schechner et al., 2003). However, it remains unclear how these plasma proteins interact with each other and in combination in relation to RBC aggregations. Some studies have reported synergetic effects on RBC aggregation in solutions comprising both fibrinogen and albumin (Ben-Ami et al., 2003; Lee et al., 2016b).

#### Measurement Techniques

Three commercially available aggregometers for the measurement of RBC aggregations include the Myrenne Aggregometer (Myrenne GmbH, Roetgen, Germany), the LORCA (R&R Mechatronics, Hoorn, Netherlands), and the RheoScan-A (Rheomeditech, Seoul, Korea). These aggregometers adopt the same principle of analyzing the syllectogram during the aggregation process but use different geometries (Baskurt et al., 2009c).

The Myrenne Aggregometer consists of a lower cone and an upper plate with infrared diode and detector (Martínez et al., 1996). Prior to testing, the sample is sheared at 500 s^–1^ for 10 s and RBC aggregates are dispersed. Then, the initial shear processing is abruptly ceased and transmitted light is measured for 10 s and the results are analyzed as stasis aggregation index, M. Another aggregation index (M1) can be measured at a low shear rate (3 *s*^–1^) instead of the stasis. The LORCA system consists of a rotating cup and a stationary bob with laser diode and two photodiodes built into the bob (Hardeman et al., 2001). A laser beam is applied onto the sample, and the backscattered light is measured with the photodetectors. The dispersing shear rate is 600 s^–1^. After abrupt stop of the shearing, backscattered light from the sample is recorded for a 120 s and then analyzed.

The RheoScan-A involves the use of a microchip consisting of a circular test chamber and a small metal stir bar. Owing to the disposable test chip, a test can be completed within 3 min (Shin et al., 2009b). When the stir bar rotates at 1000 RPM for 10 s, pre-existing RBC aggregates can be completely dispersed. After abrupt stoppage of the rotating stirrer, the transmitted light is recorded for 120 s and the light intensity-time curve is analyzed. The amplitude (*AMP*) and half-time (*T*_1__/__2_) parameters can be determined in a manner similar to other devices. The *M* index is the area below the syllectogram during the first 10 s and the *AI* refers to the ratio of the area below the syllectogram to total area during the first 10 s (Shin et al., 2009b).

### Critical Shear Stress

Conventional aggregation indexes use individually specific measurement parameters or use units of measurement that are arbitrary, such as M and AMP. Since these indexes are strongly dependent on the characteristics of specific instruments, quantitative comparisons between studies are essentially precluded. To overcome these difficulties of comparison, several studies have introduced CSS to disaggregate or disperse RBC aggregates completely. Considering various magnitudes of shear flow of *in vivo* blood circulation, the reversible dynamics of RBC aggregation and disaggregation would occur repeatedly. RBCs are disaggregated in arteries except in cases of ischemia, but RBC aggregates are easily observed in venules. Some studies have measured the critical shear rate (CSR) as the minimum shear rate to disperse RBC aggregation (Hardeman et al., 2001). However, the CSR is strongly affected by hematocrits, which effect should be corrected.

#### Measurement Techniques

**FIGURE 3:**
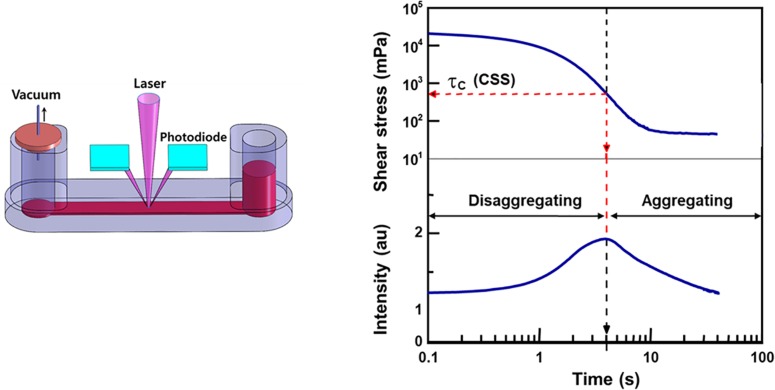
Schematic of microfluidic aggregometry to measure critical shear stress.

Given the limitations of the CSR, CSS has been used for measurement as it is hematocrit-independent (Razavian et al., 1992). Similar to the CSR, CSS is the minimum shear stress to disperse RBC aggregation. To measure CSS, one must monitor RBC aggregation while simultaneously varying a wide range of shear stresses. Due to the complex and difficult technical requirements, the CSS measurements has been delayed. Owing to development of microfluidics and optics, a transient microfluidic aggregometer was successfully developed to measure the CSS directly within 20 s (Hou and Shin, 2008; Shin et al., 2009b). The microfluidic aggregometer was further developed as a commercial instrument, named as the RheoScan-D300 (Rheomeditech, Seoul, South Korea). The disposable chip of the RheoScan-D300, as shown in Figure 3, is composed of a sample chamber holding a whole blood sample, a microchannel, and a waste chamber with a rubber lid. The whole blood is stored in a sample chamber and flown by the pressure difference through the microchannels. While the sample flows through the micro-channel, the backscattered light is measured with the photodiodes and pressure data are recorded over time. The light intensity gradually increases and then decreases. When the light intensity reach the maximum, the corresponding shear stress is defined as the critical shear-stress (*τ*_c_). The operating principles to measure CSS have been reported elsewhere (Shin et al., 2009a).

#### Factors Affecting CSS Measurements

Similar to conventional aggregation indexes, cellular and plasmatic factors affect CSS. In a previous study (Xue et al., 2013), CSS was examined with varying fibrinogen concentration as well as RBC deformability. With increasing concentrations of glutaraldehyde (GA) (0.001–0.005%) or heat treatment (HT) at 49°C, over increasingly extended time intervals (0–7 min), the RBC deformability gradually decreased, resulting in a proportional increase in CSS. The effect of cell deformability on CSS was even greater with higher concentration of fibrinogen (2–6 g/L). This indicated that there was a synergetic amplification of CSS in the presence of both reduced deformability and elevated fibrinogen levels. Both conditions of reduced RBC deformability and increased fibrinogen levels are commonly observed in diabetic patients who have microcirculatory diseases (Singh and Shin, 2009).

#### The Advantages of CSS as an RBC Aggregation Index

CSS is the minimum shear stress required to disperse RBC aggregates, and appears to be an excellent index to represent RBC aggregation. First, CSS is hematocrit-independent so hematocrit correction is not required, whereas for the CSR, the AI increases and the threshold shear-rate decreases when the hematocrit increases (Shin et al., 2009b). Second, CSS holding dimensional unit such as the millipascal (mPa) is a more physical value to compare directly with those measured with other devices than any other aggregation indexes. Most conventional aggregation indexes such as the M-index use arbitrary unit; therefore, the results obtained cannot be compared across studies. Furthermore, CSS also reflects cellular factors as well as plasmatic factors. Therefore, CSS appears to be a potentially useful index to represent RBC aggregation (Kim et al., 2013).

## Clinical Association of Hemorheology With Dn

Many studies have examined the clinical association of hemorheological alterations with DN. The clinical association of hemorheological properties with diabetic mellitus are summarized in Table 2. Since the present study considered clinical studies for human subjects, animal studies were excluded in this review.

**TABLE 2:** Clinical association of hemorheological properties with diabetic nephropathy.

	**Alteration of hemorheological properties**
Diabetic mellitus	[**Deformability**] - Impaired deformability in T2DM - Hyperglycemia-induced glycation and oxidation (Resmi et al., 2005) - formation of advanced glycation end-products (AGEs) including HbA1c - increased internal fluid viscosity & reduced membrane fluidity (Watala et al., 1985; Linderkamp et al., 1999) [**Aggregation**] - Increased aggregation in T2DM - reduced charges in RBC membrane (sialic acid moieties of glycoproteins) - increased fibrinogen level and decreased albumin in T2DM lead to synergistic increase of RBC aggregation - (Angelkort, 1999; Vayá et al., 2011; Li et al., 2015; Mahendra et al., 2015)
Diabetic Nephropathy	***[Pre-clinical studies]*** - No reports on sensitivity, specificity and ROC curve analysis - most reported decreased RBC deformability for human subjects: Kikuchi et al. (1982), [opetwcite]B97,B87[clotwcite]Zimmermann et al. (1996); Sotirakopoulos et al. (2004), Brown et al. (2005), Shin et al. (2007b), Saito et al. (2011) - rare report on RBC aggregation for human subjects - Lee et al. (2015): T2DM at 4 stages of CKD (*n* = 105), decreased RBC deformability, increased AI, CSS, fibrinogen, ACR (*p* < 0.05)
	***[Clinical studies]*** (1) Lee et al. (2018) - proposed [fibrinogen × ESR/EI], as a newly proposed diagnostic index of DN - a significant difference at all stages of DN classified according to the GFR - moderate sensitivity (74.5%), specificity (63.1%) and AUC of ROC curve (0.762) - No significant difference of RBC deformability alone to the degree of DN (2) Chung et al. (2018) - significantly higher CSS in patients with DN than in those without DN (*p* < 0.001) - CSS cut-off value: 312.67 mPa - moderate sensitivity (60.2%), specificity (60.3%) and AUC (0.635) - No significant difference of RBC deformability alone to the degree of DN

A filtration method involving meshes has been used and typical pore sizes have ranged from 3 to 5 μm. However, clinical results have varied widely depending on pore size (Tomaiuolo, 2014). Zimmermann et al. (1996) reported that RBC deformability decreased with the severity of DN and showed a high correlation with DN (*n* = 58). They found that impairment of RBC deformability associated with the concentrations of glycosylated hemoglobin in patients with T2DM. Kikuchi et al. (1982) also reported impaired RBC deformability in patients with kidney failure (*n* = 74). Sotirakopoulos et al. (2004) examined RBC deformability in patients with CKD on hemodialysis or peritoneal dialysis (*n* = 88). They found impaired erythrocyte deformability in dialysis patients and that RBC deformability was more severe immediately post-dialysis.

Two studies have been conducted using more systematic analyses. Brown et al. (2005) examined the association of impaired RBC deformability and DN using a 3 μm-pore mesh (*n* = 57). They reported that there were distinctive differences in RBC deformability between patients with diabetes mellitus without nephropathy and those with DN (*p* < 0.01). They also found a close correlation between RBC deformability and creatinine level in plasma. Saito et al. (2011) adopted the same filtration method using nickel mesh. They reported that RBC filterability for patients with diabetes was much lower than that for controls. However, erythrocyte filterability did not show any significant correlation with either the respective DN nor antidiabetic treatments.

Some studies of DN have used a microfluidic ektacytometer (RheoScan), which is suited to clinical environments. Shin et al. (2007b) investigated the association of RBC deformability with DN and DR (*n* = 212) and reported that RBC deformability of the diabetic patients with diabetes mellitus showed a significantly lower than that of control group. Moreover, for patients with complications including CKD, end-stage renal failure, DR, and for those with a combination of DR and DN, RBC deformability was further reduced when compared to patients without such diabetic complications.

Lee et al. (2015) examined various hemorheological parameters in patients with T2DM at different stages of CKD (*n* = 105), and found significant decrease in AI, deformability (EI), CSS, fibrinogen, and the ACR (all *p* < 0.05). Also, the deformability at 3 Pa (EI_@__3__Pa_) was found to be an independent predictor of GFR in multiple regression analysis. Recently, Lee et al. (2018) reported that significantly different values metabolic and hemorheological parameters were observed according to the progression of DKD, as listed Table 3. Among them, the ‘fibrinogen × ESR/EI’ showed a significant difference at the moderate CKD and severe CKD stages. With this parameter, the prevalence of microalbuminuria was diagnosed with the moderate sensitivity (74.5%) and specificity, (63.1%), respectively.

**TABLE 3:** Hemorheological parameters of a study population according to chronic kidney disease (CKD) stages.

	**CKD stage**					***P*-value**
	**Non-CKD**	**CKD 1**	**CKD 2**	**CKD 3**	**CKD 4, 5**	
		
	***n* = 340**	***n* = 31**	***n* = 21**	***n* = 52**	***n* = 10**	
Fibrinogen (mg/dL)	270.63 (56.57)	307.19^a^ (83.35)	315.76^b^ (86.86)	311.27^c^ (67.82)	357.80^d^ (77.52)	<0.001
ESR (mm/h)	14.36 (12.52)	25.29(22.21)a	27.19^b^(22.99)	31.67^c^(20.55)	51.80^d,e,f,g^(23.20)	<0.001
EI	0.3218 (0.0180)	0.3227 (0.0122)	0.3144 (0.0253)	0.3159 (0.0160)	0.3139 (0.0136)	0.048
Critical shear stress (Pa)	262.79 (131.24)	318.96 (151.73)	318.10 (194.15)	298.90 (158.84)	329.70 (83.81)	0.028
Fibrinogen/CSS	789.07 (460.60)	999.56 (526.91)	1037.17 (736.81)	953.36 (535.05)	1055.12 (284.49)	0.004
Fibrinogen/EI	807.13 (248.20)	953.92^a^ (268.55)	957.31 (314.17)	989.52^c^ (230.00)	1144.98^d^ (270.62)	<0.001
ESR/EI	45.16 (41.15)	78.12^a^ (68.39)	84.27^b^ (66.36)	100.44*c*(65.54)	165.07^d,e,f,g^(73.59)	<0.001
(Fibrinogen × ESR)/EI	13434 (15805)	26518^a^ (30729)	30997^b^ (34407)b	33872^c^ (27345)	61503^d,e,f,g^ (34934)	<0.001

*The *P-*value represents the analysis of variance P for the baseline measures among the groups. Data are presented as the mean (standard deviation). ^*a*^*p* < 0.05 between the non-CKD group and CKD group 1. ^*b*^*p* < 0.05 between the non-CKD group and CKD group 2. ^*c*^*p* < 0.05 between the non-CKD and CKD group 3. ^*d*^*p* < 0.05 between the non-CKD group and CKD groups 4,5. ^*e*^*p* < 0.05 between CKD group 1 and CKD groups 4,5. ^*f*^*p* < 0.05 between CKD group 2 and CKD groups 4,5. ^*g*^*p* < 0.05 between CKD group 3 and CKD groups 4,5. CKD, chronic kidney disease; CSS, critical shear stress; EI, elongation index at 3 Pa; ESR, erythrocyte sedimentation rate (reproduced with permission from Lee et al., 2018).*

Chung et al. (2018) had conducted a similar clinical study using the same instrument as Lee et al. (2018). However, they reported somewhat different results in relation to DN (*n* = 421). CSS was much higher in patients with DN than in those without DN (317.43 ± 125.11 vs. 385.22 ± 182.89, *p* < 0.001) (Table 4). After considering some factors including age, sex and diabetic duration, the highest tertile of CSS showed three folds the risk of DKD compared to compared to the lowest CSS tertile. With a CSS cut-off value (312.7 mPa), the estimated GFR (eGFR) was also moderately predicted with 60.3% of sensitivity and 59.6%, specificity. Also, uACR was diagnosed with 60.2% of sensitivity and 60.3% of specificity.

**TABLE 4:** Baseline characteristics of patients based on the estimated glomerular filtration rate (eGFR) (*n* = 421).

	**eGFR(mL/min/l.73m^2^)**		
	**>60 (*n* = 345)**	**<60 (*n* = 76)**	***P*-value**
Sex(M:F)	I.5:1	1.30:1	0.588
Age (Yrs)	56.36 ± 11.11	66.03 ± 9.85	< 0.001
BMI (Kg/m^2^)	24.99 ± 7.47	23.84 ± 3.43	0.070
Diabetes duration (Yrs)	7.16 ± 7.22	13.69 ± 9.37	< 0.001
HTN(n(%))	180 (53.4)	62 (82.7)	< 0.001
FPG(mg/dL)	165.64 ± 52.34	176.35 ± 67.27	0.299
HbAlc(%)	8.19 ± 2.02	8.77 ± 2.67	0.098
HOMA-IR	4.52 ± 3.79	4.51 ± 2.50	0.981
HOMA-B	48.19 ± 37.72	46.89 ± 46.92	0.860
Hb (g/dL)	14.37 ± 1.54	12.51 ± 1.86	< 0.001
T-Cho (mg/dL)	181.27 ± 44.23	173.26 ± 55.29	0.241
HDL-Cho (mg/dL)	51.76 ± 13.91	47.10 ± 15.54	0.020
LDL-Cho (mg/dL)	96.12 ± 38.66	90.04 ± 46.38	0.299
TG(mg/dL)	169.61 ± 110.06	184.16 ± 114.05	0.322
ESR(mm/H)	20.01 ± 20.83	40.67 ± 28.80	< 0.001
hsCRP (mg/dl.)	0.28 ± 0.71	0.57 ± 1.30	0.099
Fibrinogen (mg/dL)	311.21 ± 54.78	338.44 ± 58.57	0.018
CS5(mPa)	317.43 ± 125.11	385.22 ± 182.89	< 0.001
EI@3Pa (%)	30.60 ± 1.91	30.42 ± 20.54	0.481
Fibrinogen/EI@3Pa (mg/dI.%)	1033.28 ± 197.76	1141.44 ± 268.71	0.045
DR(%)	32.0	46.5	0.019
DPN(%)	15.4	29.3	0.004
CAD(%)	8.4	23.7	< 0.001

*BMI, body mass index; CAD, coronary artery disease; CSS, critical shear stress; DPN, diabetic peripheral neuropathy; DR, diabetic retinopathy; EI@3Pa, elongation index at 3 pascal; ESR, erythrocyte sedimentation rate; FPG, fasting plasma glucose; Hb, hemoglobin; HbA1c, glycated hemoglobin; HDL/LDL-cho, high/low density lipoprotein cholesterol; HOMA-IR/B, homeostasis model assessment of insulin resistance/beta-cell function; hs-CRP, high sensitivity c-reactive protein; HTN, hypertension; T-cho, total-cholesterol; TG, triglyceride (reproduced under CC BY 4.0 with permission from Chung et al. (2018).*

In these two recent studies (Chung et al., 2018; Lee et al., 2018), RBC deformability did not show a significant difference according to the degree of DN. This was an unexpected result given that RBC deformability has been proposed as a potential index of diabetic microangiopathy for more than two decades. Lee et al. (2015) had earlier proposed the use of a fibrinogen EI, and later reported that a fibrinogen × ESR/EI showed slightly higher sensitivity than the fibrinogen EI in predicting DN among various hemorheological markers (Lee et al., 2018). Fibrinogen, which is elevated in patients with diabetes, has been associated with RBC aggregation (Vayá et al., 2011). The ESR, commonly known as an indicator of inflammation, has also been found to associate with RBC aggregation in diabetic patients (Li et al., 2015). Therefore, the fibrinogen × ESR/EI, as a combined index of hemorheological factors, has been recently proposed in diagnosing DN (Lee et al., 2018). However, in Chung et al. (2018), the fibrinogen EI was marginally higher for the eGFR in a group aged < 60 years old, whereas CSS alone showed a significant difference between patients with diabetes with and without DN.

As noted, CSS is significantly affected by fibrinogen concentration and cell deformability (Xue et al., 2013). Thus, CSS may provide the same information as the fibrinogen × ESR/EI. CSS is another index of RBC aggregation; however, CSS has a crucial advantage in that it does not need to adjust hematocrit, unlike the conventional aggregation indices (Shin et al., 2009a). Also, CSS holds the dimensional unit such as mPa, which enables to direct comparison of the measured CSS values between different instruments (Lee et al., 2016a). Similar to whole blood viscosity, CSS values vary with temperature (Lim et al., 2010). Therefore, CSS may be a potential index to diagnose DKD.

## Conclusion and Future Perspectives

Diabetes mellitus is a growing burden on global healthcare. Diabetes-related complications have led to an increasing mortality rate. DKD is a main diabetic complication, occurring in patients with a long duration of diabetes. DKD occurs due to the dysfunction of glomerular microvasculature of the kidney. DKD is a clinical syndrome characterized with persistent albuminuria, which can be identified through observing a continuous decline in the GFR. The uACR is a common method to screen for DN; however, the uACR is influenced in relation to various factors including a patient’s general dietary condition, medications, comorbidities, and improper urinary sampling (Wu et al., 2014). It is suggested to confirm the diagnosis of microalbuminuria through repeated urine tests over a period of 3–6 months (Nazar, 2014).

Hemorheological alteration has been suggested as a potential biomarker of DN for more than three decades. Most studies have reported that cell deformability is generally reduced in patients with DKD, but findings suggest that it is not sufficiently precise to use for patient screening. However, more recent studies have reported that cell deformability alone did not show a high correlation with DKD. Instead, two other parameters have been proposed to diagnose DKD or DN. One involved a combination of hemorheological parameters, namely, the fibrinogen × ESR/EI (Lee et al., 2018), and the other used CSS (Chung et al., 2018). Both parameters represent reduced deformability, increased aggregation of RBCs, and elevated fibrinogen concentration. Furthermore, the effects of these parameters has been considered in relation to whole blood, and also showed a close correlation with DN (Monica et al., 2019). However, whole blood viscosity is complex, with hemorheological parameters affected due to various factors non-related to diabetes mellitus. A careful combination of hemorheological parameters directly related to DN would more likely lead to a successful diagnosis of DN.

CSS is not only associated with diabetes-related micro-vascular complications but also diabetes-related macro-vascular complications. Park et al. (2018) reported that CSS was strongly correlated with acute myocardial infarction in patients with T2DM. CSS has also been found to correlate highly with hyperlipidemia (Gyawali et al., 2015a), chronic inflammatory indexes, and oxidative stress (Gyawali et al., 2015b). Furthermore, Gyawali et al. (2014) reported that CSS alone could be used to diagnose metabolic syndrome (MetS) with higher sensitivity and specificity than other conventional indexes such as the ankle brachial pressure index. The cut-off value of CSS for diagnosing DKD was 310 mPa (Chung et al., 2018), whereas the mean CSS in healthy controls was 200.5 mPa (Shin et al., 2009a). Cho et al. (2014) reported a 30% increase in CSS in acute coronary syndrome: 265 mPa in patients with stable angina, 338 mPa in patients with unstable angina, and 324 mPa in patients with acute myocardial infarction. Therefore, CSS can be considered as a potential index to diagnose not only DKD but also other diabetes-related complications. More research can be valuable in confirming the relationship between elevated CSS and diabetic vascular complications and finding each relevant cut-off value to use as a screening tool.

## Author Contributions

HL, WN, SL, CA, JM, KW, and SS contributed to the literature survey, brainstorming, writing, and critical review of the manuscript. HL, WN, and SS edited the manuscript.

## Conflict of Interest Statement

The authors declare that the research was conducted in the absence of any commercial or financial relationships that could be construed as a potential conflict of interest.

## Abbreviations

ACR: albumin-to-creatinine ratio
AGEs: advanced glycation end-products
AI: aggregation index
AUC: area under the curve
CKD: chronic kidney disease
CSR: critical shear-rate
CSS: critical shear stress
DKD: diabetic kidney disease
DN: diabetic nephropathy
DPN: diabetic peripheral neuropathy
DR: diabetic retinopathy (DR)
EI: elongation index
ESR: erythrocyte sedimentation rate
GFR: glomerular filtration rate
POC: point-of-care
T2DM: type 2 diabetes mellitus.
